# Staple Line Reinforcement During Laparoscopic Sleeve Gastrectomy: Systematic Review and Network Meta-analysis of Randomized Controlled Trials

**DOI:** 10.1007/s11695-022-05950-z

**Published:** 2022-02-16

**Authors:** Alberto Aiolfi, Michel Gagner, Marco Antonio Zappa, Caterina Lastraioli, Francesca Lombardo, Valerio Panizzo, Gianluca Bonitta, Marta Cavalli, Giampiero Campanelli, Davide Bona

**Affiliations:** 1 Department of Biomedical Science for Health, Division of General Surgery, Istituto Clinico Sant’Ambrogio, University of Milan, Via Luigi Giuseppe Faravelli, n16, 20149 Milan, Italy; 2 Hôpital du Sacre Coeur, Quebec Montreal, Canada; 3 UOC Chirurgia Generale Ospedale Fatebenefratelli, Asst Fatebenefratelli-Sacco Milano, Milan, Italy

**Keywords:** Laparoscopic sleeve gastrectomy, Staple line reinforcement, Suture reinforcement, Oversewing

## Abstract

**Purpose:**

Staple line reinforcement (SLR) during laparoscopic sleeve gastrectomy (LSG) is controversial. The purpose of this study was to perform a comprehensive evaluation of the most commonly utilized techniques for SLR.

**Materials and Methods:**

Network meta-analysis of randomized controlled trials (RCTs) to compare no reinforcement (NR), suture oversewing (SR), glue reinforcement (GR), bioabsorbable staple line reinforcement (Gore® Seamguard®) (GoR), and clips reinforcement (CR). Risk Ratio (RR), weighted mean difference (WMD), and 95% credible intervals (CrI) were used as pooled effect size measures.

**Results:**

Overall, 3994 patients (17 RCTs) were included. Of those, 1641 (41.1%) underwent NR, 1507 (37.7%) SR, 689 (17.2%) GR, 107 (2.7%) GoR, and 50 (1.3%) CR. SR was associated with a significantly reduced risk of bleeding (RR=0.51; 95% CrI 0.31–0.88), staple line leak (RR=0.56; 95% CrI 0.32–0.99), and overall complications (RR=0.50; 95% CrI 0.30–0.88) compared to NR while no differences were found vs. GR, GoR, and CR. Operative time was significantly longer for SR (WMD=16.2; 95% CrI 10.8–21.7), GR (WMD=15.0; 95% CrI 7.7–22.4), and GoR (WMD=15.5; 95% CrI 5.6–25.4) compared to NR. Among treatments, there were no significant differences for surgical site infection (SSI), sleeve stenosis, reoperation, hospital length of stay, and 30-day mortality.

**Conclusions:**

SR seems associated with a reduced risk of bleeding, leak, and overall complications compared to NR while no differences were found vs. GR, GoR, and CR. Data regarding GoR and CR are limited while further trials reporting outcomes for these techniques are warranted.

**Graphical abstract:**

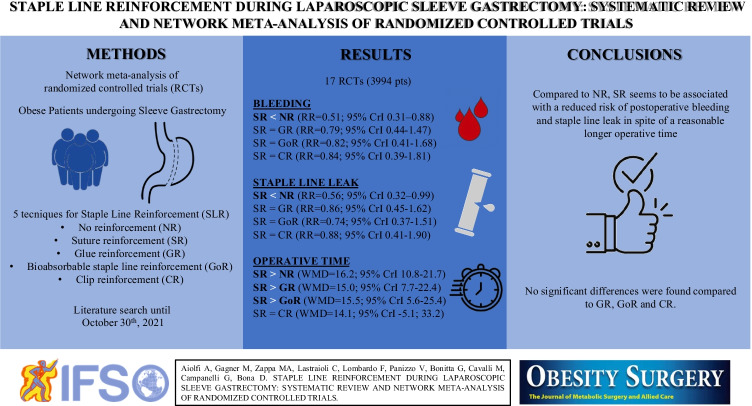

**Supplementary Information:**

The online version contains supplementary material available at 10.1007/s11695-022-05950-z.

## Introduction

Laparoscopic sleeve gastrectomy (LSG) has gained increasing worldwide recognition since its excellent results in terms of weight loss and over long-term sustained comorbid resolution [1–3]. Compared with other bariatric surgeries, it has many advantages such as technical simplicity, lack of anastomoses, feasible bridge treatment in high-risk patients, lower major morbidity (0.2–10%), and mortality (<1%) [4–6]. For all these reasons, LSG has become the most common bariatric for morbid obesity all around the world [7–10].

Early staple line complications (SLC), such as bleeding and leak, may occur and their incidence may vary from 1 to 6% [11, 12]. Results can be clinically devastating for the patient and expensive for the facility with prolonged hospitalization and resources utilization. In attempt to reduce the incidence of early SLC, different techniques for staple line reinforcement (SLR) have been described [13, 14]. Suture oversewn (SR), reinforcement with glue (GR), bioabsorbable staple line reinforcement (Gore® Seamguard®) (GoR), and clipping (CR) have been reported. By contrast, there are many surgeons who chose to not reinforce the staple lines (NR) either for concern over the costs and/or the lack of proven benefits [15]. Many studies have been published to assess the impact of various reinforcement techniques on the prevention of early SLC [16–19]. However, outcomes were heterogeneous because of the retrospective design, limited sample sizes, and low postoperative complication rates. Similarly, previously published meta-analyses gave conflicting and heterogeneous conclusions because of the inclusion of observational studies. Furthermore, different reinforcement techniques were grouped and considered together in the pairwise comparison thus making unfeasible a real subgroup analysis [20–23]. Therefore, a robust consensus supported by low heterogeneity with a comprehensive consideration of all techniques is lacking.

Hence, the aim of this study was to perform a network meta-analysis to provide a comprehensive evidence on efficacy of NR, SR, GR, GoR, and CR on early SLC in the setting of randomized controlled trials (RCTs).

## Materials and Methods

A systematic review was performed following the Preferred Reporting Items for Systematic Reviews and Network Meta-Analyses (PRISMA-NMA) checklist guidelines [24]. Institutional review board approval was not required. MEDLINE, Scopus, Web of Science, Cochrane Central Library, and ClinicalTrials.gov were used [25]. The last date of search was October 30th, 2021. A combination of the following MeSH terms (Medical Subject Headings) were used (“sleeve gastrectomy” (tiab), OR “vertical gastrectomy” (tiab)) AND (“reinforcement” (tiab), OR “staple line reinforcement” (tiab)) AND (“bleeding” (tiab), OR “hemorrage” (tiab)) AND (“leak” (tiab), OR “leakage” (tiab)). All titles were initially evaluated and suitable abstracts were then extracted. The study protocol was registered at the international prospective register of systematic reviews (PROSPERO Registration Number: CRD42021286380).

### Eligibility Criteria

Inclusion criteria: (a) RCTs comparing surgical outcomes for NR, SR, GR, GoR, and CR in the setting of elective LSG; (b) when two or more papers were published by the same institution, study group, or used the same dataset, articles with the longest follow-up or the largest sample size; (c) in case of duplicate studies with accumulating numbers of patients, only the most complete reports were included for quantitative analysis. Exclusion criteria: (a) articles were not written in English; (b) the study methodology or surgical technique was not clearly reported; (c) studies reporting mixed data including other surgical approaches (i.e., gastric bypass, and gastric banding); (d) studies non-reporting any of the a priori defined primary outcomes; (e) studies reporting outcomes for re-do surgery; (f) studies with <15 patients per treatment-arm.

### Data Extraction

The following data were collected: authors, year of publication, country, study design, number of patients, sex, age, body mass index (BMI), American Society of Anesthesiologists (ASA), comorbidities, surgical technique for staple line reinforcement, and postoperative surgical outcomes. All data were computed independently by three investigators (AA, CL, AS) and compared at the end of the reviewing process. A fourth author (DB) reviewed the database and determined discrepancies.

### Outcomes of Interest

Primary outcomes were postoperative bleeding and staple line leak within a short-term (90-day) assessment. Secondary outcomes were sleeve stenosis, surgical site infection (SSI), reoperation, estimated intraoperative blood loss (ml), operative time (OT) (minutes), hospital length of stay (HLOS) (days), overall complications, and 30-day mortality. Postoperative bleeding was defined as significant early postoperative hemodynamic changes, including one or more of the following: an increase in heart rate >20 beats/min, a decrease in blood pressure >20 mmHg, a drop in hemoglobin >3 g/dl, transfusion requirement, or signs of active or recent bleeding on CT scan [26]. Staple line leak was classified according to the modified UK Surgical Infection Study Group definitions [27]. Leakage was confirmed by extravasation of the contrast material outside the gastric tube on an abdominal computed tomography (CT) scan and suspected in case of collections near the gastric tube confirmed during repeat laparoscopy or upper gastrointestinal endoscopy. Surgical site infection (SSI) was defined as the development of superficial, deep, or organ/space SSI in accordance with the Center for Disease Control and Prevention guidelines (https://www.cdc.gov/nhsn/opc/ssi/Accessed on October 15^th^ 2021).

### Quality Assessment

Three authors (AA, CL, AS) independently assessed the methodological quality of selected trials by using the Cochrane risk of bias tool [28]. This tool evaluates the following criteria: (1) method of randomization; (2) allocation concealment; (3) baseline comparability of study groups; and (4) blinding and completeness of follow-up. Trials were graded as having low (green circle), high (red circle), or unclear (yellow circle) risk of bias.

### Statistical Analysis

We performed a fully Bayesian arm-based random effect network meta-analysis [29–31]. Compared to the frequentist meta-analysis, the Bayesian approach takes into account all sources of variation, reflects these variations in the pooled result, can provide accurate estimates for small samples, and allows computation of predictive distribution [32, 33]. We used risk ratio (RR) as a pooled effect size measure for categorical outcomes and weighted mean difference (WMD) for continuous outcomes. Related to RR, we adopted a “sceptical” prior distribution with mean and scale equal to 0 and 0.4 (10% of the distribution is contained within the clinically unimportant null interval). A consistency generalized linear model as described in the NICE-DSU technical document was fitted [34]. We assigned normal distribution with zero mean and scale 100 as vague prior distribution. Assuming a common heterogeneity parameter across the treatment comparisons, we used an informative half-normal prior with zero mean and scale 0.5 or the between-study variability (*τ*) [35]. Sensitivity analysis regarding the choice of prior distribution for *τ* was considered [36]. The local inconsistencies were investigated using the node split, but was not possible to conduct a formal assessment of the consistency of the direct and indirect evidence where the evidence network included open loops. Statistical heterogeneity (*I*^2^ index) was evaluated: value of 25% or smaller was defined as low heterogeneity, value between 50 and 75% as moderate heterogeneity, and 75% or larger as high heterogeneity [37]. The inference was performed using mean and relative 95% credible intervals (CrI). We consider the estimated parameter statistical significant when its 95% CrI encompasses null hypothesis value [38]. The plot of leverage values vs. the square root of the residual deviance was used to identify potential outlier. The transitivity assumption was considered and descriptive statistics were generated to compare the distributions of baseline participant characteristics across studies and treatment comparisons. The treatment ranking probability indicates which approach is the best in dependence of a given outcome. The confidence in estimates of the outcome was assessed using Confidence in Network Meta-Analysis (CINeMA) [39]. Statistical analyses were performed using JAGS and R-Cran 3.4.3 (Distributed Statistical Computing; Vienna, Austria) [40, 41].

## Results

### Systematic Review

**Fig. 1:**
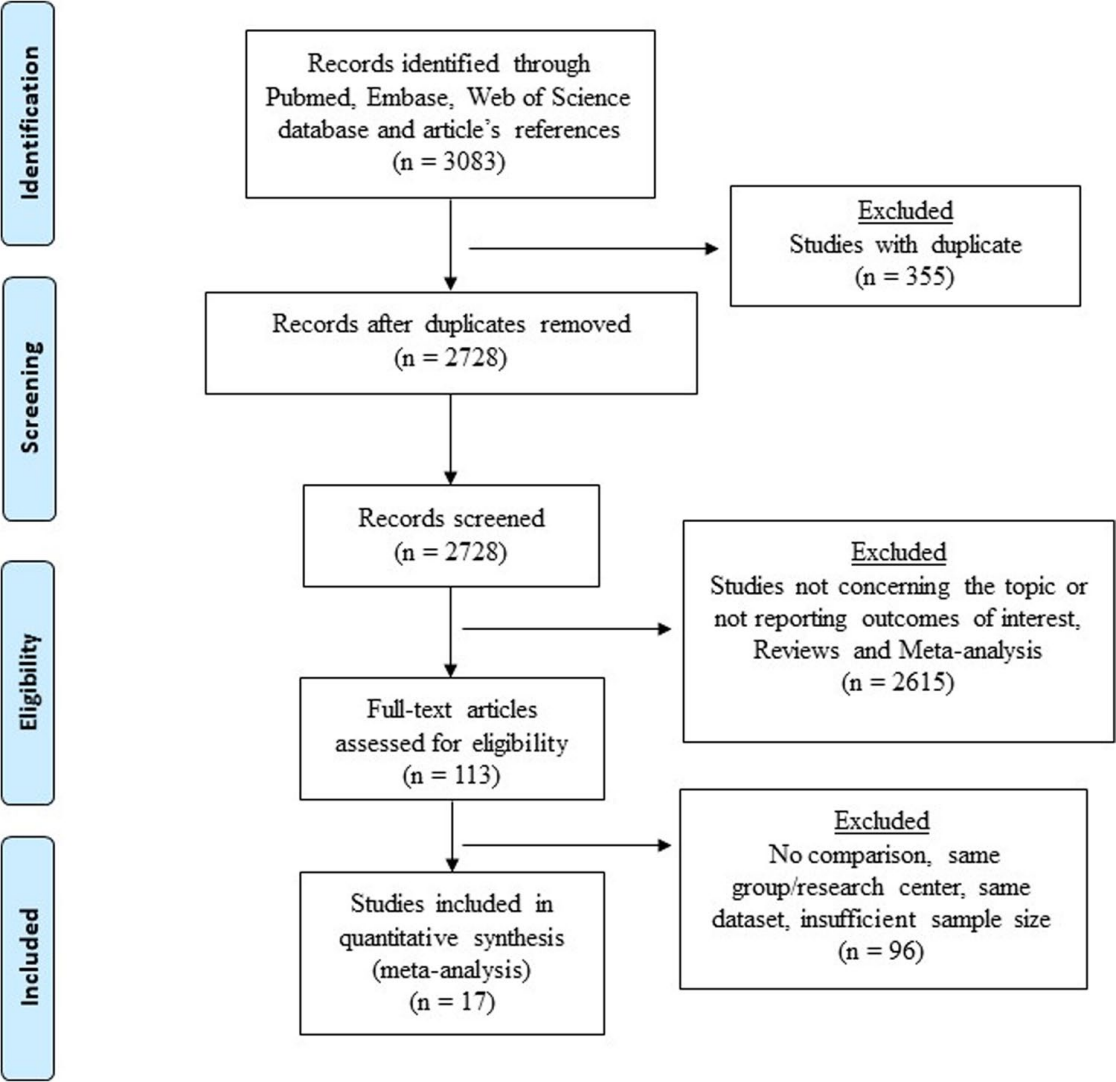
The Preferred Reporting Items for Systematic Reviews and Network Meta-Analyses (PRISMA-NMA) checklist diagram

The selection process flow chart is reported in Figure 1. Our initial search identified 3083 publications. After removing duplicates, 2728 titles and abstracts were reviewed. Further screening found 17 RCTs meeting the predefined inclusion/exclusion criteria. The included RCTs had issues regarding blinding taking into consideration that the application of blinding into surgical RCTs is challenging (Supplementary Figure 1). The method of randomization was reported in 12 studies while 6 RCTs described the operating surgeon’s proficiency. Details regarding the power analysis were specified in 5 studies, 1 study was underpowered, and 12 studies do not report these data (Supplementary Table 1).

Overall, 3994 patients were included for the analysis. Of those, 1641 (41.1%) underwent NR, 1507 (37.7%) SR, 689 (17.2%) GR, 107 (2.7%) GoR, and 50 (1.3%) CR (Table 1). The patient’s age ranged from 28 to 51 years and the majority were female (63.9%). The BMI ranged from 38.2 kg/m^2^ to 54.1 kg/m^2^ and the ASA score was reported in 4 studies. There was no evidence of violation of the transitivity assumption, based on the observations that the common treatment (NR) was consistent across trials, effect modifiers were equally distributed across studies, and participants could in principle be randomized to any of the treatments being compared in the network. Finally, the design-by-treatment interaction model showed no evidence of statistically significant inconsistency (*P*=0.693). Descriptive statistics for all outcomes are reported in Table 2.

**Table 1:** Demographic and clinical characteristics of patients undergoing NR (no reinforcement), SR (suture staple line oversewing), GR (glue reinforcement), GoR (bioabsorbable staple line reinforcement; Gore® Seamguard®), and CR (clips reinforcement). *Yrs* years, *BMI* body mass index, *ASA score* American Society of Anesthesiologists Classification, *OT* operative time, *HLOS* hospital length of stay, *Nr* not reported. Data are reported as numbers, mean ± standard deviation, and median (range).

**Author, year, country**	**Study period**	**Techniques**	**No. of patients**	**Mean age (yrs)**	**Sex ratio (F/M)**	**Mean BMI (kg/m**^**2**^**)**	**Mean ASA**	**Mean OT (min)**	**Mean HLOS (days)**
Dapri et al., 2009, France [42]	2008–2009	NR	25	44.3	15/10	44.2 ± 6.3	2.5 ± 0.5	47.4 ± 10.7	3.6±1.4
		GoR	25	39.4	14/11	49.7 ± 7	2.4 ± 0.5	48.9 ± 18.4	3.9±1.5
		SR (PDS®)*	25	41.3	8/17	47.7 ± 10.5	2.5 ± 0.5	59.9 ± 19.6	2.8±0.8
Albanopoulos et al., 2011, Greece [43]	2009–2010	GoR	48	37.6	29/19	46.08	nr	55.3 ± 4.1	3.9 ±2.1
		SR (PDS®)	42	37.9	24/18	47.4	nr	69.4 ± 3.8	3.3 ±1.1
Musella et al., 2011, Italy [44]	2007–2011	SR (Prolene®)*	50	33.9 ± 10.4	25/15	49.6 ± 2.9	2.02	89 ± 4	nr
		NR	50	33.3 ± 10.1	18/22	48.9 ± 3.1	2.14	80 ± 4	nr
Gentileschi et al., 2012, Italy [45]	2010–2011	SR	35	44.6± 9.6	35/5	47.2 ± 6.3	nr	84 ± 4.6	nr
		GoR	34	44.1 ± 8.3	34/6	47± 6.7	nr	64 ± 3.2	nr
		GR (Floseal®)	33	44.1 ± 10.3	33/7	47.4 ± 6.5	nr	66 ± 3.2	nr
Aggarwal et al., 2013, India [46]	2009–2011	SR (PDS®)*	30	38.3	21/9	49.8	nr	139 ± 10	4.3 ±0.9
		NR	30	37.5	19/11	49.3	nr	117 ± 19	4.4 ±0.9
Bülbüller et al., 2013, Turkey [47]	2012–2013	NR	15	35.2	9/6	49.2	nr	138.1	nr
		SR (Prolene®)*	16	35.6	10/6	50.2	nr	196.0	nr
		SR (V-loc®)*	16	39.6	10/6	48.3	nr	166.4	nr
		GR (Fibrin)	18	36.2	11/7	48.7	nr	138.2	nr
Musella et al., 2014, Italy [48]	2009-nr	GR (Fibrin)	50	32.3 ± 9.9	14/36	43.6 ± 4.4	2.48	82.8 ± 5.2	5.1 ± 1.1
		NR	50	33.2 ± 7.9	21/29	44.8 ± 8.5	2.34	84.3 ± 6.2	5.2 ± 1.2
Shah et al., 2014, India [49]	2011–2012	SR (PDS®)	51	39.2 ± 14.6	51/30	46.1 ± 8.5	nr	58.8±19.7	nr
		NR	49	36 ± 11.6	49/16	44.7 ± 9.8	nr	72.8±25.8	nr
Albanopoulos et al., 2015, Greece [50]	2012–2013	SR (PDS®)*	84	38 ± 13	52/32	46.9 ±7.6	2.2±1.3	45± 21	4.0 ±3.75
		NR	62	36 ± 17	18/44	45.65 ±6.6	2.3±1.2	40±20	3.5±2.0
Sroka et al., 2015, Israel [51]	2013–2014	GR (Evicel®)	49	39.6 ± 10.15	35/14	43.11 ± 5.87	nr	64±23	nr
		SR (PDS®)*	49	35.9 ± 11.6	34/15	42.13 ± 4.46	nr	74±21	nr
		NR	67	38 ± 12.6	43/24	43.82 ± 5.32	nr	54±19	nr
Carandina et al., 2016, France [52]	2012–2014	NR	150	39.3 ± 11.3	123/27	43.3 ± 5.1	nr	100.7±16.4	5.7±1.1
		GR (Evicel®)	150	37.2 ± 11.1	123/27	43 ± 5.7	nr	104.5±22.1	6.1±6.1
		SR (Monocryl) *	150	35.5 ± 11.2	118/32	44 ± 13	nr	126.2±18.9	6±4.6
		SR (V-loc®)*	150	37.1± 11.7	120/30	43.8 ± 10.7	nr	124.6±22.8	6.1±4
Kwiatkowski et al., 2016, Poland [53]	2014–2015	SR (Biosyn®)*	50	36.8 ± 10.3	27/23	49 ± 8.5	nr	78.2 ± 20.5	3.2 ±0.4
		CR	50	39.5 ± 10.5	19/24	45.7 ± 9	nr	64.1 ± 16.5	3.6 ±1.3
Alamdari et al., 2018, Iran [54]	2015–2016	NR	100	32.8±4.2	62/38	44.61±5.8	nr	52.03±8.1	nr
		SR (PDS®)*	99	31.6±3.7	58/42	44.97±6.6	nr	69.64±9.6	nr
Hany et al., 2018, Egypt [55]	2016–2017	SR (V-loc®)*	460	37.8 ± 11.8	207/153	47.64 ± 7.29	nr	69 ± 1.65	1.92 ± 0.33
		NR	460	38.0 ± 11.15	293/167	47.07 ± 7.38	nr	50.8 ± 1.58	1.97 ± 0.42
Rebibo et al., 2018, France [56]	2014–2017	GR (Fibrin)	293	40.1± 10.7	216/77	44.6 ± 5.5	nr	56.7 ± 16.3	1
		NR	293	38.9 ± 10.3	215/78	44.5 ± 5.5	nr	55.9 ±14.4	1
Taha et al., 2018, Egypt [57]	2014–2016	SR (Vycril) *	200	33.9 ± 9.2	117/83	42.4 ± 4.4	nr	51.3 ± 4.3	2.5 ± 1.2
		NR	200	33.5± 9.6	113/87	42.4 ± 4.3	nr	44.3± 5.5	2.6 ± 0.9
Pilone et al., 2019, Italy [58]	2017-nr	GR (Glubran 2®)	96	37.4 ± 3.5	52/44	44.6 ± 4.1	nr	83.3	4.5 ± 1.5
		NR	90	39.6 ± 5	58/32	45.7 ± 3.8	nr	75.3	5.8 ± 2.0

*Running/continuous suture

**Table 2:** Descriptive statistics stratified according to different treatment. NR (no reinforcement), SR (suture staple line oversewing), GR (glue reinforcement), GoR (bioabsorbable staple line reinforcement; Gore® Seamguard®), and CR (clips reinforcement). *HLOS* hospital length of stay, *SSI* surgical site infections. Values are presented as percentages (range) for categorical variables and as mean (range) for continuous variables

	**CR (*n*=50)**	**GoR (*n*=107)**	**GR (*n*=689)**	**NR (*n*=1641)**	**SR (*n*=1507)**
**Categorical outcomes**					
**Postoperative bleeding**	4.0 (4.0–4.0)	1.87 (0.0–2.94)	1.6 (0.0–6.12)	3.72 (0.0–16.0)	1.1 (0.0–8.0)
**Staple line leak**	2.0 (2.0–2.0)	3.74 (0.0–8.0)	0.87 (0.0–3.0)	1.64 (0.0–6.67)	0.59 (0.0–6.25)
**Sleeve stenosis**	2.0 (2.0–2.0)	3.74 (0.0–8.0)	0.87 (0.0–3.03)	1.64 (0.0–6.67)	0.59 (0.0–6.25)
**Reoperation**	4.0 (4.0–4.0)	1.87 (0.0–4.0)	0.29 (0.0–3.03)	0.06 (0.0–4.08)	0.04 (0.0–6.25)
**Morbidity**	6.0 (6.0–6.0)	1.87 (0.0–2.94)	3.05 (1.36–6.12)	5.85 (0.0–20.0)	1.77 (0.0–10.0)
**30-day mortality**	0.0 (0.0–0.0)	0.0 (0.0–0.0)	0.29 (0.0–2.0)	0.0 (0.0–0.0)	0.07 (0.0–6.25)
**SSI**	-	0.0 (0.0–0.0)	0.22 (0.0–2.04)	1.01 (0.0–10.0)	0.62 (0.0–4.0)
**Continuous outcomes**					
**Operative time (minutes)**	64.1 (64–64)	56.6 (48.9–64)	75.8 (56.7–138)	61.1 (40–138)	76.6 (45–196)
**HLOS (days)**	3.6 (3.6–3.6)	3.9 (3.9–3.9)	3.22 (1.0–6.1)	2.79 (1.0–5.8)	2.99 (1.92–6.0)

### Network Meta-analysis

#### Primary Outcomes

**Fig. 2:**
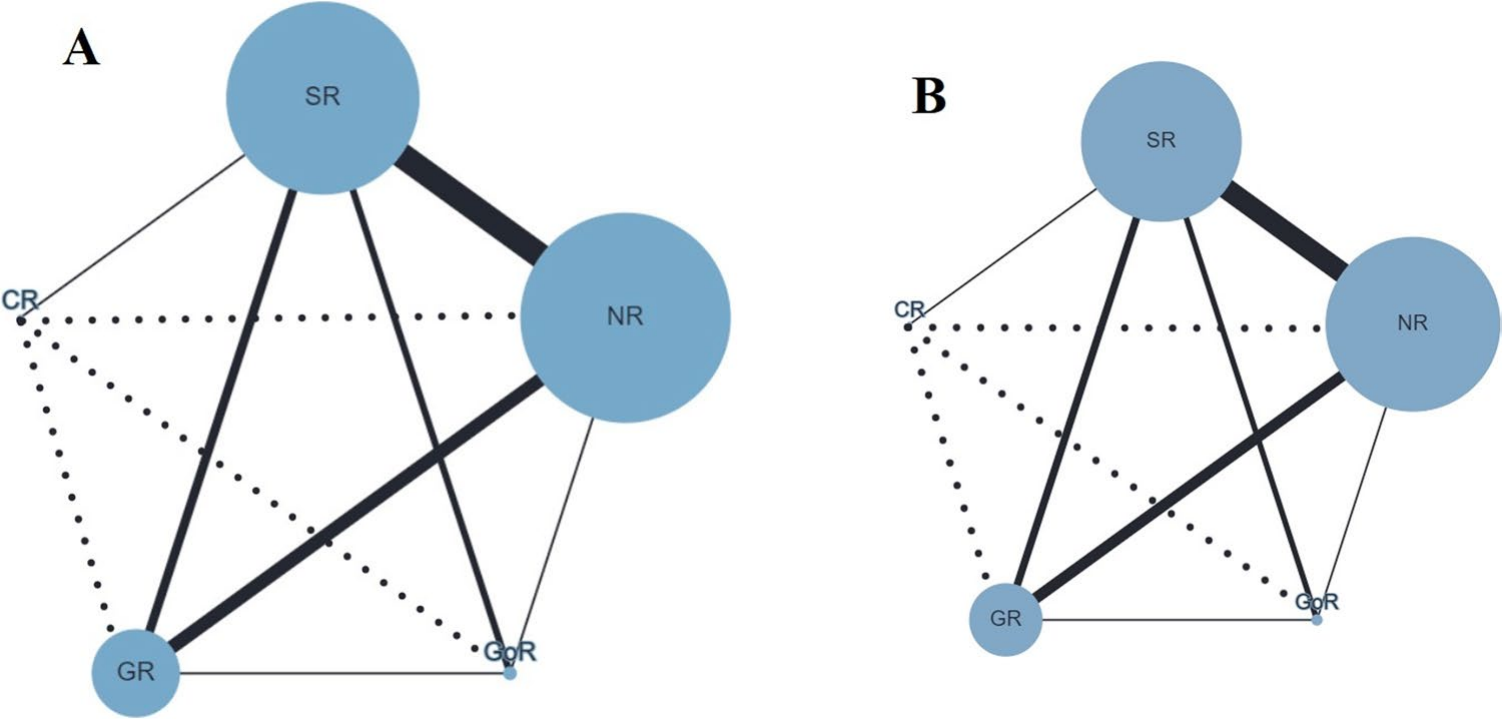
Network geometry for primary outcomes: **A** postoperative bleeding, **B** staple line leak. Node size reflects the sample size while edges width reflects the number of studies for a specific pairwise comparison. Continuous lines represent direct comparisons while dashed lines represent indirect comparisons obtained with the network analysis. No reinforcement (NR), suture reinforcement (SR), glue reinforcement (GR), bioabsorbable staple line reinforcement (Gore® Seamguard®) (GoR), and clips reinforcement (CR)

Seventeen studies (3994 patients) [42–58] reported postoperative bleeding (Figure 2A). SR was associated with a significantly reduced postoperative bleeding compared to NR (RR=0.51; 95% CrI 0.31–0.88) while no significant differences were found for SR vs. GR (RR=0.79; 95% CrI 0.44–1.47), SR vs. GoR (RR=0.82; 95% CrI 0.41–1.68), and SR vs. CR (RR=0.84; 95% CrI 0.39–1.81). The global heterogeneity was low (*I*^2^=8.2%; 95% CrI 0.0–23.7%). Staple line leak was reported in 17 studies (3994 patients) [42–58] (Figure 2B). SR was associated with a significantly reduced risk of leak compared to NR (RR=0.56; 95% CrI 0.32–0.99). No significant differences were found for SR vs. GR (RR=0.86; 95% CrI 0.45–1.62), SR vs. GoR (RR=0.74; 95% CrI 0.37–1.51), and SR vs. CR (RR=0.88; 95% CrI 0.41–1.90). The global heterogeneity was zero (*I*^2^=0.0%; 95% CrI 0.0–23.3%). The treatment ranking evaluation graded SR as the surgical approach with the lowest probability to be ranked as first treatment for high postoperative bleeding (21.3%) and staple line leak (22.8%).

#### Secondary Outcomes

SR was associated with a significantly longer operative times (17 studies; 3994 patients) [42–58] compared to NR (WMD=16.2; 95% CrI 10.8–21.7), GR (WMD=15.0; 95% CrI 7.7–22.4), and GoR (WMD=15.5; 95% CrI 5.6–25.4) while no significant differences were found for the comparison with CR (WMD=14.1; 95% CrI–5.1; 33.2). Sleeve stenosis was reported in 7 studies (1692 patients) [44, 46, 48, 50, 52, 53, 56]. No significant differences were found for SR vs. NR (RR=1.09; 95% CrI 0.55–2.17), SR vs. GR (RR=0.77; 95% CrI 0.37–1.59), and SR vs. CR (RR=0.88; 95% CrI 0.41–1.91). SSI (10 studies; 2733 patients) [42, 43, 47, 49–51, 55, 56] was similar for SR vs. NR (RR=0.67; 95% CrI 0.37–1.33), SR vs. GR (RR=0.76; 95% CrI 0.32–1.81), and SR vs. GoR (RR=0.72; 95% CrI 0.27–1.98). Postoperative complication was reported in 17 studies (3994 patients) [42–58]. SR was associated with a significantly reduced risk compared to NR (RR=0.50; 95% CrI 0.30–0.88) while no significant differences were found for SR vs. GR (RR=0.98; 95% CrI 0.55–1.76), SR vs. GoR (RR=0.84; 95% CrI 0.42–1.71), and SR vs. CR (RR=0.88; 95% CrI 0.38–1.75). Hospital length of stay (17 studies; 3994 patients) [42–58] and 30-day mortality (17 studies; 3994 patients) [42–58] were comparable among treatments. The league table for all outcomes with both direct and indirect comparisons is reported in Table 3. The node split analysis did not show evidence against inconsistency. The sensitivity analysis showed robustness of findings. The leverage plots do not show evidence of study outliers into this network meta-analysis.

**Table 3:** League table. NR (no reinforcement), SR (suture staple line oversewing), GR (glue reinforcement), GoR (bioabsorbable staple line reinforcement; Gore® Seamguard®), and CR (clips reinforcement). Each row represents a specific outcome. Values in each column represent the relative effect of the referral treatment (bold) with the comparator. Values are expressed as risk ratio (RR) and 95% credible intervals (95%CrI) for categorical outcomes and as weighted mean difference (WMD) and 95% credible intervals (95%CrI) for continuous outcomes. *I*^*2*^ heterogeneity

**Categorical variables**					**I**^**2**^ **(95%CrI)**	**Outcomes**
**CR**	1.02 (0.37–2.87)	1.06 (0.41–2.78)	1.69 (0.67–4.14)	0.84 (0.39–1.8)	8.2 (0.0–18.3)	Postoperative bleeding
0.98 (0.35–2.73)	**GoR**	1.04 (0.42–2.57)	1.65 (0.7–3.82)	0.82 (0.41–1.68)		
0.94 (0.36–2.47)	0.96 (0.39–2.38)	**GR**	1.59 (0.8–3.02)	0.79 (0.44–1.47)		
0.59 (0.24–1.49)	0.61 (0.26–1.43)	0.63 (0.33–1.25)	**NR**	0.5 (0.31–0.88)		
1.19 (0.56–2.55)	1.22 (0.6–2.42)	1.27 (0.68–2.29)	2.02 (1.13–3.28)	**SR**		
**CR**	1.18 (0.42–3.35)	1.02 (0.38–2.74)	1.56 (0.62–1.06)	0.82 (0.41–1.90)	0.0 (0.0–23.3)	Staple line leak
0.84 (0.30–2.36)	**GoR**	0.86 (0.35–2.14)	1.32 (0.56–3.09)	0.74 (0.37–1.50)		
0.97 (0.36–2.61)	1.16 (0.47–2.99)	**GR**	1.52 (0.74–3.18)	0.86 (0.46–1.63)		
0.64 (0.25–1.62)	0.76 (0.32–1.78)	0.66 (0.31–1.35)	**NR**	0.56 (0.33–0.99)		
1.13 (0.53–2.42)	1.34 (0.66–2.68)	1.16 (0.61–2.18)	1.77 (1.01–3.05)	**SR**		
**CR**	1.15 (0.40–3.27)	0.81 (0.29–2.28)	0.89 (0.41–1.91)	-	0.0 (0.0–27.4)	Sleeve stenosis
0.87 (0.31–2.50)	**GR**	0.704 (0.28–1.83)	0.77 (0.38–1.60)	-		
1.24 (0.44–3.43)	1.42 (0.55–3.57)	**NR**	1.09 (0.55–2.17)	-		
1.13 (0.52–2.43)	1.29 (0.63–2.65)	0.91 (0.46–1.81)	**SR**	-		
**CR**	1.16 (0.41–3.24)	1.0 (0.38–2.79)	1.06 (0.41–2.72)	0.84 (0.40–1.77)	0.0 (0.0–22.3)	Reoperation
0.86 (0.31–2.45)	**GoR**	0.87 (0.33–2.32)	0.92 (0.37–2.31)	0.72 (0.35–1.50)		
0.99 (0.36–2.66)	1.15 (0.43–3)	**GR**	1.06 (0.47–2.37)	0.83 (0.43–1.60)		
0.94 (0.37–2.41)	1.08 (0.43–2.71)	0.94 (0.42–2.14)	**NR**	0.78 (0.43–1.42)		
1.12 (0.57–2.53)	1.38 (0.67–2.83)	1.20 (0.62–2.32)	1.28 (0.70–2.28)	**SR**		
**GoR**	1.04 (0.37–2.92)	1.38 (0.52–3.65)	0.93 (0.43–2.02)	-	0.0 (0.0–32.1)	SSI
0.96 (0.34–2.67)	**GR**	1.32 (0.56–3.09)	0.89 (0.44–1.79)	-		
0.72 (0.27–1.92)	0.77 (0.32–1.80)	**NR**	0.67 (0.37–1.25)	-		
1.07 (0.49–2.3)	1.12 (0.56–2.25)	1.49 (0.80–2.67)	**SR**	-		
**CR**	0.98 (0.35–2.76)	0.84 (0.32–2.18)	1.64 (0.65–4.04)	0.83 (0.39–1.76)	26.4 (14.3–39.5)	Postoperative complications
1.02 (0.36–2.87)	**GoR**	0.86 (0.35–2.10)	1.67 (0.69–3.90)	0.84 (0.42–1.71)		
1.19 (0.46–3.09)	1.17 (0.48–2.85)	**GR**	1.96 (0.97–3.67)	0.98 (0.55–1.76)		
0.61 (0.25–1.53)	0.60 (0.26–1.44)	0.51 (0.27–1.03)	**NR**	0.50 (0.30–0.88)		
1.21 (0.57–2.56)	1.19 (0.58–2.40)	1.02 (0.57–1.81)	1.99 (1.13–3.27)	**SR**		
**CR**	1.19 (0.41–3.47)	1.36 (0.48–3.83)	1.04 (0.38–2.92)	0.934 (0.43–2.01)	0.0 (0.0–26.1)	30-day mortality
0.84 (0.29–2.46)	**GoR**	1.15 (0.41–3.16)	0.88 (0.33–2.38)	0.79 (0.37–1.66)		
0.73 (0.26–2.08)	0.87 (0.32–2.44)	**GR**	0.76 (0.32–1.91)	0.68 (0.34–1.39)		
0.96 (0.34–2.65)	1.14 (0.42–3.06)	1.31 (0.53–3.16)	**NR**	0.89 (0.46–1.74)		
1.07 (0.50–2.31)	1.27 (0.60–2.67)	1.46 (0.72–2.96)	1.12 (0.57–2.17)	**SR**		
**Continuous variables**					***I***^**2**^ **(95%CrI)**	**Outcomes**
**CR**	−1.40 (−22.98; 20.09)	−0.95 (−21.6; 19.54)	−2.07 (−22.07; 17.8)	14.09 (−5.043; 33.24)	97.8 (82.3–100)	Operative time (minutes)
1.40 (−20.09; 22.98)	**GoR**	0.48 (−10.75; 11.68)	−0.67 (−11.18; 9.83)	15.49 (5.66; 25.45)		
0.95 (−19.54; 21.6)	−0.48 (−11.68; 10.75)	**GR**	−1.15 (−8.15; 5.81)	15.04 (7.68; 22.42)		
2.07 (−17.8; 22.07)	−0.67 (−9.83; 11.18)	1.15 (−5.81; 8.15)	**NR**	16.2 (10.85; 21.57)		
−14.09 (−33.2; 5.04)	−15.49 (−25.45; −5.66)	15.04 (−22.42; −7.68)	16.2 (−21.57; −10.85)	**SR**		
**CR**	0.32 (−1.10; 1.71)	−0.61 (−1.95; 0.74)	−0.31 (−1.53; 0.92)	−0.40 (−1.53; 0.73)	72.9 (59.3–85.6)	Hospital stay (days)
−0.32 (−1.71; 1.10)	**GoR**	−0.92 (−1.98; 0.17)	−0.63 (−1.53; 0.29)	−0.72 (−1.55; 0.14)		
0.60 (−0.74; 1.95)	0.92 (−0.17; 1.98)	**GR**	0.29 (−0.29; 0.88)	0.20 (−0.53; 0.94)		
0.31 (−0.92; 1.53)	0.63 (−0.29; 1.53)	−0.29 (−0.88; 0.29)	**NR**	−0.09 (−0.56; 0.38)		
0.40 (−0.73; 1.53)	0.72 (−0.14; 1.55)	−0.20 (−0.94; 0.53)	0.09 (−0.38; 0.56)	**SR**		

## Discussion

There are several technical methods for SLR during LSG. Up to date, a definitive consensus regarding the benefits of one technique over another is missing while a robust evidence-based indication is lacking. Our results suggest that compared to NR, SR may be associated with a reduced risk of postoperative bleeding, leak, and overall complications in spite of a reasonable longer operative time. No significant differences among treatments were found in terms of sleeve stricture, SSI, risk of reoperation, and 30-day mortality.

Over the last decades, LSG has become a worldwide approved weight loss bariatric operation [10]. The procedure involves a mostly vertical stapled transection of the stomach, removal of the gastric fundus, and proximal antrum to create a tubular alimentary channel along the lesser curvature [5]. Food restriction, early satiety, decreased ghrelin production, and increased production of GLP-1 and PYY-36 have been reported [59, 60]. According to a recent meta-analysis, LSG has been associated with 57.6% mean excess weight loss (EWL) at 1 year and 70.1% EWL at 3 years [1]. Principal advantages of LSG include its relative procedural simplicity, absence of anastomosis, maintenance of gastrointestinal continuity, no or low risk of ulceration and internal hernia, lower rates of dumping syndrome, lowered ghrelin levels, and improved quality of life [9, 61, 70. ]. The overall complication rate in referral centers is <15% while in-hospital mortality is around 0.3% [6, 62]. Specifically, early SLC such as bleeding and staple line leak may occur up to 6% of patients [11, 12, 63]. These SLC are associated with an increased mortality risk with higher hospital costs and resources utilization [64]. Therefore, even a small reduction in early SLC could prevent serious events such as reoperation, blood transfusions, peritonitis, and septic shock with a presumed benefit on global costs.

The incidence of postoperative bleeding has been reported ranging from 1.1 to 8.7% [45, 65]. In line with literature, our results seem to reflect previously reported data ranging from 1.1 to 4.0% (Table 2). In our study, SR was associated with a significantly reduced risk of postoperative bleeding compared to NR (RR=0.51; 95% CrI 0.31–0.88) while no significant differences were found in the comparison with other treatments. However, despite the lack of statistical significance, the point estimation of SR compared with other treatments was below 1.00 thus possibly suggesting a trend toward a clinical benefit (Table 3). This is in line with D’Ugo et al. that, in recent multicenter retrospective study, reported lower bleeding rates for staple line oversewing compared to no reinforcement (1.4% vs. 13.7%; *p*=0.02) [66]. Similarly, Sroka et al. found that suture oversewing minimized hemorrhagic complications with reasonable operative time prolongation [51]. In contrast, Choi et al. [20] did not found any significant benefit for staple line suture oversewing while other authors argued that suture oversewing could potentially increase the risk bleeding because of tissue tearing at the point of stitch penetration [67]. Interestingly, the treatment ranking evaluation graded SR as the surgical approach with the lowest probability to be ranked as first treatment for high postoperative bleeding while the global heterogeneity was low 8.2%. These findings add robustness to the result. However, even if the majority of bleeding events after LSG derive from the gastric staple line, it should be considered that the omentum, short gastric vessels, spleen, gastroepiploic artery, and abdominal wall may constitute other possible sources of oozing. Therefore, despite the low heterogeneity, our results should be interpreted cautiously as potentially influenced by operating surgeon experience and expertise, learning curve, patient comorbidities (i.e., hypertension and chronic renal failure), postoperative drugs use (i.e., ketorolac and heparinoids), technical tricks (i.e., use of bipolar or unipolar electrocautery devices), type of suture (interrupted vs. continuous), and thickness of laparoscopic staplers. Finally, the type of suture material (absorbable vs. nonabsorbable), sewing technique (baseball stitch, simple oversewing, locking, imbricating, etc.), and extension of staple line oversewing (entire staple line vs. selected regions) may potentially constitute other sources of bias and should be assessed in future RCT.

The incidence of staple line leak after LSG has been previously reported ranging from 0.5 to 2.7% [13]. In our study, the pooled quantitative results for staple line leak ranged from 0.5 to 3.7% (Table 2). Interestingly, SR was associated with a reduced risk of staple line leak compared to NR (RR=0.56; 95% CrI 0.32–0.99). This result should be interpreted extremely cautiously because the 95% CrI upper limit approaches the non-significant limit (1.00). Therefore, future studies and large sample size are mandatory to deeply assess this issue. Again, despite the lack of statistical significance, the point estimation of SR compared with other treatments seems to suggest a clinical trend toward reduced staple line leak (Table 3). This is in line with Aggarwal et al. [46] that reported a reduced leak rate in patients that underwent staple line oversewing. Notably, no significant differences were found for the comparison SR vs. GoR. This is similar to what reported by Albanopoulos et al. [43] and Gentileschi et al. [45] that described similar postoperative leak rates comparing staple line oversewing and reinforcement with bioabsorbable reinforcement material (Gore® Seamguard®). Interestingly, despite the lack of statistically significant differences for the comparison GoR vs. NR, the point estimation was below 1.00 thus possibly suggesting a trend toward an improved clinical benefit for GoR. This is in line with what reported by Gagner et al. in a recent systematic review [13]. These results may be influenced by the limited patient population in the GoR (*n*=107) and CR (*n*=50) group. Therefore, these initial suggestions should be considered with caution while future and further analyses are needed to corroborate these preliminary findings. The treatment ranking evaluation graded SR as the surgical approach with the lowest probability to be ranked as first treatment for high postoperative leak (21.3%) and the global heterogeneity was 0.0%. However, it should be considered that despite the low heterogeneity, several factors such as patient age, comorbid conditions, BMI, ASA score, smoking status, smaller bougie size, distance of the transection from the pylorus, hospital protocols, implementation of enhanced recovery after surgery protocols (ERAS), and surgeons’ experience may constitute source of bias. The included RCTs reported only data for bioabsorbable staple line reinforcement (Gore® Seamguard®); therefore, further RCT are required in the future to deeply assess the role of other bioabsorbable reinforcement materials (i.e., bovine pericardium) [13, 15].

Interestingly, SR was found to be associated with a reduced risk of postoperative complications compared to NR (RR=0.50; 95% CrI 0.30–0.88) while no differences were found in the comparison with other treatments. This is similar to what reported by Wang et al. [23]. This effect may be driven by the reduced risk of postoperative bleeding and staple line leak. The global heterogeneity was moderate (*I*^2^=26.4%); therefore, this result should be interpreted with caution because possibly influenced by patients’ comorbidities, preoperative patients’ selection, BMI, antibiotic therapy, ASA grade, smoke status, surgical technique, and surgeon experience [68]. Finally, differently from Albanopoulos et al. [43] that reported higher rate of sleeve stenosis after oversewing, we were not able to find significant differences in terms of sleeve stenosis, after SR. A longer operative time was found for SR vs. NR (WMD=16.2 min), SR vs. GR (WMD=15.0 min), and SR vs. GoR (WMD=15.5 min). This time prolongation seems reasonable and related to the adjunctive procedure performed after gastric stapling. These data are comparable with a previous study that concluded that oversewing the staple line during LSG determines an extra operative time of 14.4 min (range 8–18) [45, 69]. This may influence overall costs because of the increased utilization of materials and operative room occupation [64]. However, it should be considered that the reduced postoperative bleeding and postoperative complications may mitigate the initial expenses with ultimate global cost-effectiveness. A dedicated cost analysis was not feasible in our study as the majority of the included studies did not report financial data. Therefore, further studies are required to deeply assess this issue.

The surgeons’ performance with different levels of training and experience might have impact on patient outcomes and can be a significant source of bias. It has been shown that these operator-related factors are of outmost importance for determining operative time, blood loss, and overall complications. In the present meta-analysis, 11 studies do not report specific data about the surgeon that performed the procedure while 6 trials described the operating surgeons’ proficiency. The included RCT data were reported from high-volume teaching hospitals; therefore, results should be interpreted carefully and may not be applicable to small non-teaching hospitals. Therefore, this meta-analysis also intends to plea for further qualitative and standardized RCTs to address the type of suture (absorbable vs. non absorbable), the type of sewing (single suture vs. continuous, etc.), the role of other bioabsorbable reinforcement materials (i.e., bovine pericardium), and related costs.

To our knowledge, this is the first systematic review and network analysis that includes all RCTs of this topic that have been published up to date. Using network meta-analytical techniques, we were able to globally synthesize data from numerous studies and therefore rank the treatments. The study was planned in agreements with PRISMA guidelines, and followed a rigorous methodology that was a priori stated in the PROSPERO protocol. This included comprehensive outcome measures and the evaluation of quality at study level (risk of bias) and confidence in results at outcome level (CINeMA). The selection criteria led to a homogenous population for the two primary outcomes, as confirmed by low heterogeneity.

There are some limitations to the current analysis. First, although transitivity assumption was met with no evidence of statistically significant inconsistency in the network analysis, the accuracy of our results can be tempered by differences in operating surgeon proficiency with a possible confounding effect on bleeding, leak rates, and postoperative complications. Second, even though only RCTs were included for our analyses, the quality of evidence remained moderate, in part, due to no blinding of patients and/or surgeons, limited power in some trials, different methods for randomization, and quality control. Specifically, the assessments of confidence in the estimates using CINeMA show moderate to very low confidence, essentially due to study limitation and imprecision. Third, as surgeries were performed by expert surgeons at high-volume referral centers, results may not be generalizable. Fourth, there was no uniformity in the surgical technique with differences in the choice of the stapler, staple cartridge, and bougie size depending on operating surgeon preference. Fifth, definitions and outcomes reporting may be different among included studies; however, it may be presumed that these disparities would be equally distributed across treatment groups. Sixth, the number of patients in the GoR and CR groups was limited. Lastly, efficacy in terms of postoperative percentage excess weight loss and co-morbidity resolution was not assessed because data were lacking.

## Conclusions

There are several technical methods for staple line reinforcement during LSG. Compared to NR, SR seems to be associated with a reduced risk of postoperative bleeding, staple line leak, and overall complications in spite of a reasonable longer operative time. No significant differences were found in terms of sleeve stricture, SSI, risk of reoperation, and 30-day mortality among all treatments. Data regarding GoR and CR are still limited; therefore, further trials reporting outcomes for these surgical techniques are necessary. As the overall quality of included RCTs was narrow because of issues regarding blinding, methods of randomization, and operating surgeon proficiency, further well-designed appropriate powered trials are warranted to corroborate our findings.

## Supplementary information

Supplementary Table 1 (DOCX 18 kb)

Supplementary Figure 1 Risk of bias for Randomized Controlled Trials (RCT) was assessed with use of the Cochrane risk-of-bias tool. Green circle: Low risk of Bias. Red circle: High Risk of Bias. Yellow circle: Unclear Risk of Bias. (PNG 994 kb)

High resolution image (TIF 6311 kb)
