# Supplementary material for: Staple Line Reinforcement During Laparoscopic Sleeve Gastrectomy: Systematic Review and Network Meta-analysis of Randomized Controlled Trials
Source: Obes Surg. 2022 Feb 16;32(5):1466–78. doi: 10.1007/s11695-022-05950-z (PMC8986671; doi:10.1007/s11695-022-05950-z)
Supplement: Supplementary file 1 — (DOCX 18 kb) [file 11695_2022_5950_MOESM1_ESM.docx]

| **Author,**  **Year, Country** | **Study design** | **Method of randomization** | **Surgeons' eligibility** | **Surgical quality control** | **Blinding** | **Power analysis** |
| --- | --- | --- | --- | --- | --- | --- |
| Dapri et al.,  2009, France ^(43)^ | nr | randomly permuted blocks of sizes 6 and 9 | nr | nr | N | N |
| AlbaNpoulos et al., 2011, Greece ^(44)^ | nr | random number table | nr | nr | P: blind.  S: blind. | nr |
| Musella et al.,  2011, Italy ^(45)^ | comparative RCT | nr | nr | nr | nr | Y |
| Gentileschi et al.,  2012, Italy ^(46)^ | comparative RCT | shuffling method | single experienced surgeon | nr | nr | N |
| Aggarwal et al.,  2013, India ^(47)^ | nr | computer-generated random numbers | nr | nr | N | nr |
| Bülbüller et al.,  2013, Turkey ^(48)^ | nr | nr | nr | nr | N | nr |
| Musella et al.,  2014, Italy ^(49)^ | nr | nr | nr | nr | nr | N |
| Shah et al.,  2014, India ^(50)^ | nr | block randomization. block size: 6. | nr | nr | nr | Y (U) |
| AlbaNpoulos et al., 2015, Greece ^(51)^ | nr | computer-generated random numbers | >400 LSG | nr | N | Y |
| Sroka et al.,  2015, Israel ^(52)^ | Non-inferiority  RCT | nr | >500 LSG | nr | N | Y |
| Carandina et al.,  2016, France ^(53)^ | nr | computer-based Nn-stratified randomization | experienced surgeons | nr | P: blind.  S: blind. | N |
| Kwiatkowski et al., 2016, Poland ^(54)^ | comparative RCT | block randomization | nr | nr | nr | N |
| Alamdari et al.,  2018, Iran ^(55)^ | nr | simple random allocation technique | nr | nr | N | nr |
| Hany et al.,  2018, Egypt ^(56)^ | comparative RCT | block 1:1 randomization. Block size: 8 | experienced surgeons | nr | P: blind. S:blind. | Y |
| Rebibo et al.,  2018, France ^(57)^ | Non-inferiority RCT | computer-based stratified 1:1 randomization, stratified by: center, age, sex, gender, and BMI | nr | nr | nr | N |
| Taha et al.,  2018, Egypt ^(58)^ | nr | computer generated, allocation concealment by sealed envelopes | nr | nr | P:blind | nr |
| Pilone et al.,  2019, Italy ^(59)^ | Non-inferiority RCT | nr | experienced surgeons | nr | nr | N |

**Supplementary Table 1**. Randomized Clinical Trials (RCTs) quality evaluation. LSG: laparoscopic sleeve gastrectomy. P: patient. S: surgeon. Y: yes. N: No. UP: under-powered. nr: not reported.
