# Supplementary figures and images for: Staple Line Reinforcement During Laparoscopic Sleeve Gastrectomy: Systematic Review and Network Meta-analysis of Randomized Controlled Trials
Source: Obes Surg. 2022 Feb 16;32(5):1466–78. doi: 10.1007/s11695-022-05950-z (PMC8986671; doi:10.1007/s11695-022-05950-z)

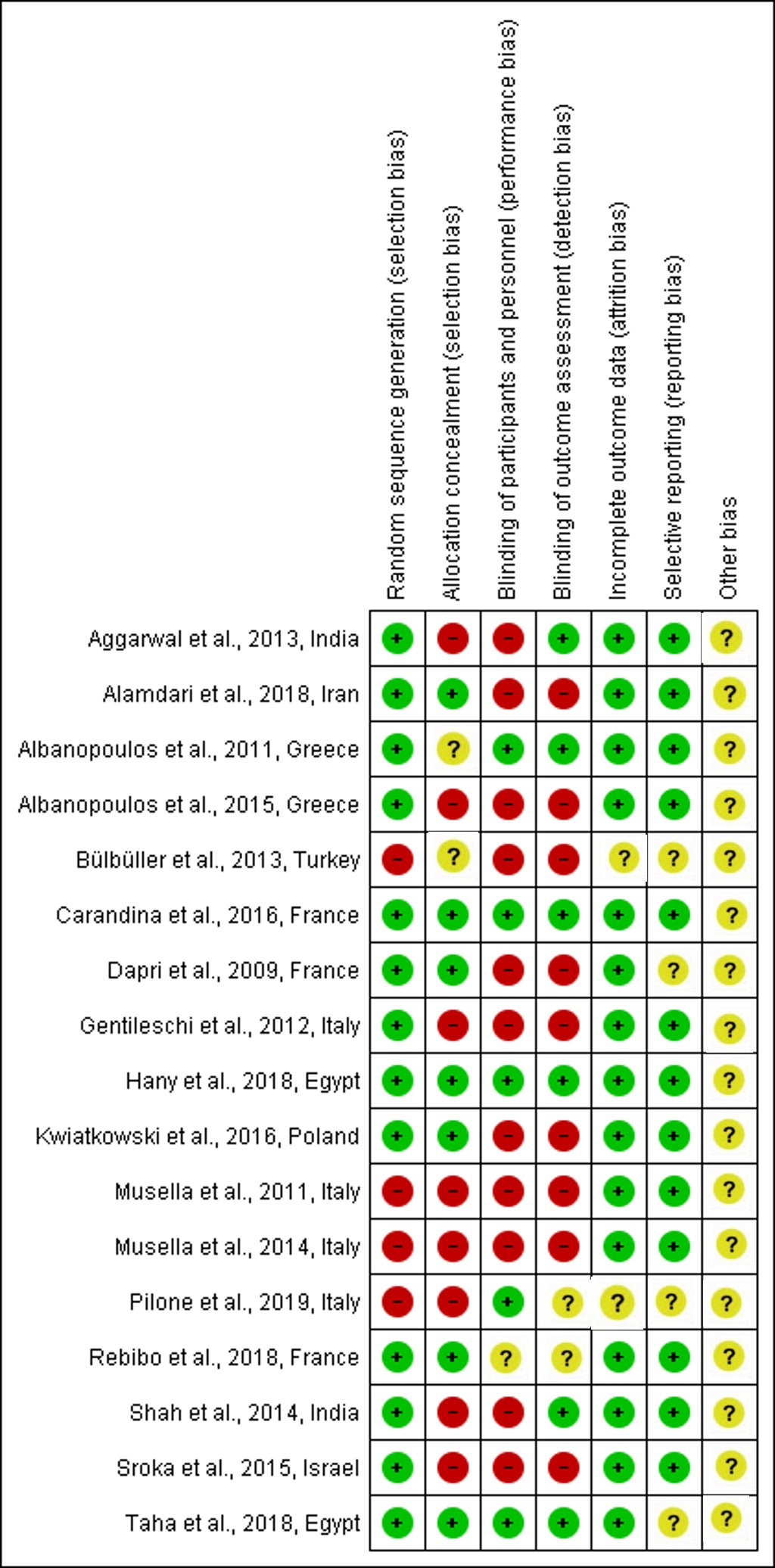

Supplement: Supplementary file 2 — Risk of bias for Randomized Controlled Trials (RCT) was assessed with use of the Cochrane risk-of-bias tool. Green circle: Low risk of Bias. Red circle: High Risk of Bias. Yellow circle: Unclear Risk of Bias. (PNG 994 kb) [file 11695_2022_5950_Fig3_ESM.png]

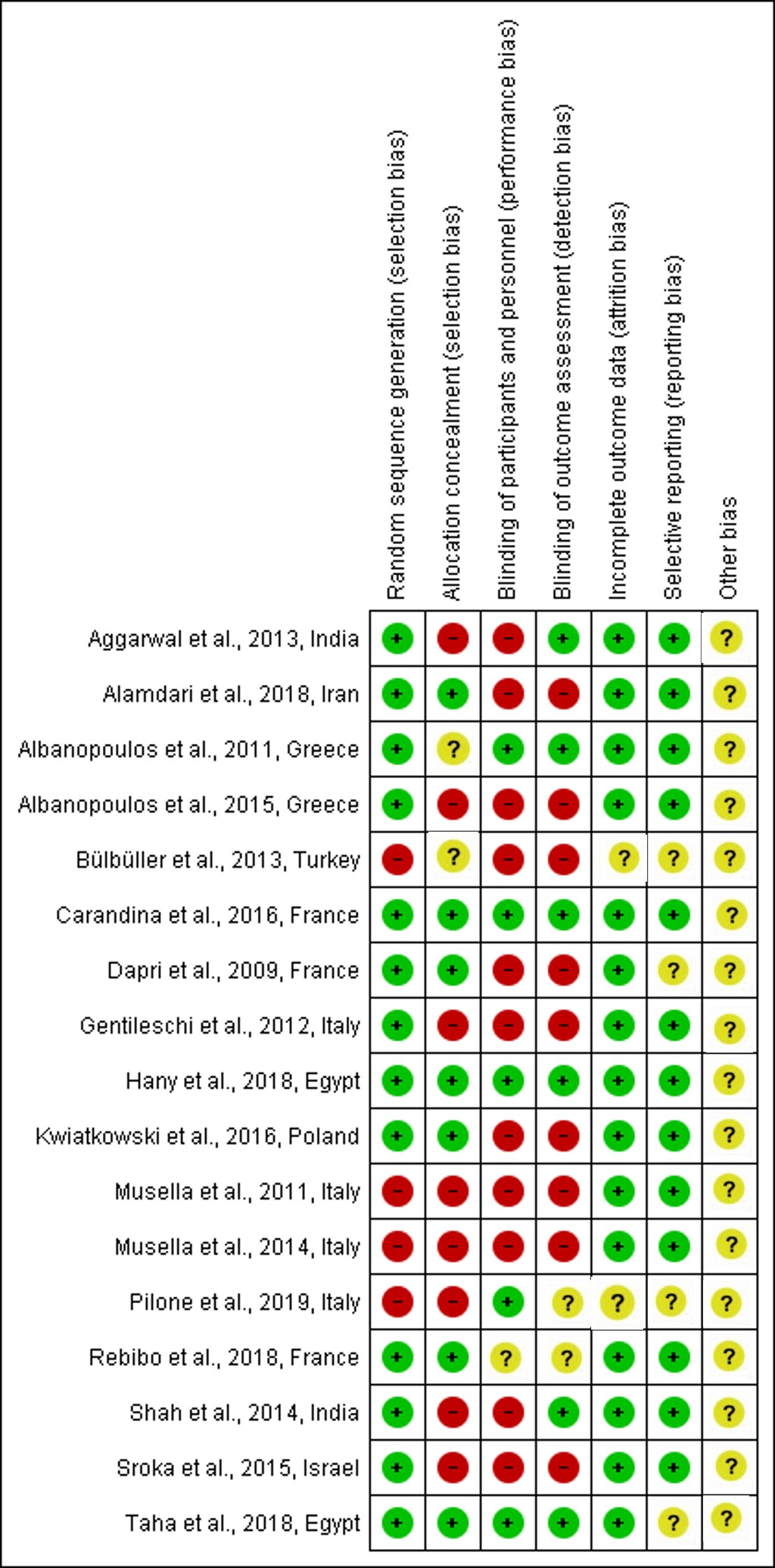

Supplement: Supplementary file 3 — High resolution image (TIF 6311 kb) [file 11695_2022_5950_MOESM2_ESM.tif]
